# Spain’s Rising Melanoma Threat: A Comprehensive 30-Year Analysis (1990–2019)

**DOI:** 10.3390/cancers16061167

**Published:** 2024-03-15

**Authors:** Lucía Cayuela, José-Juan Pereyra-Rodríguez, Juan-Carlos Hernández-Rodriguez, Aurelio Cayuela

**Affiliations:** 1 Department of Internal Medicine, Hospital Severo Ochoa, 28914 Leganés, Spain; lcayuela@salud.madrid.org; 2 Department of Medicine, School of Medicine, University of Seville, 41004 Seville, Spain; 3 Department of Dermatology, Virgen del Rocío University Hospital, 41013 Seville, Spain; juanc.hernandez.sspa@juntadeandalucia.es; 4 Unit of Public Health, Prevention and Health Promotion, South Seville Health Management Area, 41014 Seville, Spain; aurelio.cayuela.sspa@juntadeandalucia.es

**Keywords:** melanoma, incidence trends, age–period–cohort analysis, Spain, public health

## Abstract

**Simple Summary:**

Spain faces a growing concern with rising melanoma cases, affecting people of all ages and sexes. This study explores the reasons behind this trend, examining the influence of factors affecting entire populations over time and differences between age groups. Sunlight exposure remains the primary culprit, potentially linked to historical changes in sunbathing habits. This research highlights a need for improved public health strategies, emphasizing sun protection and early detection measures, especially tailored to address the specific challenges observed in Spain.

**Abstract:**

Aim: This study aims to elucidate the factors driving melanoma incidence trends in Spain by analyzing the GBD-2019 dataset (1990–2019) and investigating the age-specific, birth cohort, and period effects on incidence rates. Materials and Methods: This study analyzed melanoma incidence trends in Spain from 1990 to 2019 using an ecological design. Data were sourced from the Global Burden of Disease Study 2019 and Spain’s National Statistics Institute. Age-standardized incidence rates (ASIRs) were calculated using joinpoint regression analysis, and age–period–cohort (A-P-C) modeling was employed to assess the effects of age, time period, and birth cohort on incidence rates. Results: Between 1990 and 2019, an estimated 147,823 melanoma cases were diagnosed in Spain. The ASIRs showed a steady increase for both sexes, with slightly higher rates observed in men. Both men (average annual percentage change (AAPC): 2.8%) and women (AAPC: 2.4%) showed a steady increase in the ASIR over the period. Joinpoint analysis revealed distinct periods of incidence rate changes, with significant upward trends in earlier years followed by stabilization in recent years. Incidence rates increased steadily with age, with the highest rates in the 80–84 age group. Women tended to have slightly higher rates in younger age groups, while men had higher rates in older age groups. Both men and women experienced a steady increase in relative risk of melanoma throughout the 30-year study period, with significant upward trends across birth cohorts. Conclusions: Despite limitations, this study provides valuable insights into factors influencing melanoma incidence in Spain. By understanding age, period, and cohort effects, effective prevention strategies can be developed to reduce melanoma incidence.

## 1. Introduction

The growing global burden of melanoma is a major public health challenge [1]. There were an estimated 331,649 new cases and 58,644 deaths in 2022 [2], and projections point to an alarming increase to 510,000 cases and 96,000 deaths in 2040 [3].

Melanoma mortality trends in Spain present a unique puzzle. While rates rose sharply between the mid-1970s and early 1990s [4], more recent data show a surprising stabilization and even a decline in certain age and sex groups [5,6]. However, projections indicate a future increase in deaths, particularly among older adults, suggesting a possible increase in overall mortality and age-standardized rates for older people [7].

Despite extensive research on mortality, studies analyzing melanoma incidence data in Spain remain scarce and focused on specific regions [8,9,10,11,12]. This critical gap hinders our understanding of the true national burden and limits the development of effective prevention strategies, resource allocation, and patient care.

The Global Burden of Disease (GBD) study provides a valuable resource, meticulously tracking various diseases and injuries worldwide, including melanoma [13]. Thanks to the GBD study, detailed melanoma incidence data exist for numerous regions and countries [14,15,16,17,18]. However, Spain lacks comparable analyses, creating a significant gap in understanding the nationwide burden of this disease. This limited data availability hinders effective prevention strategies, resource allocation, and optimal patient care.

Previous studies using GBD data, including those incorporating data from Spain, have reported upward trends in melanoma cases across various geographical areas and countries [17]. However, these analyses primarily relied on age-standardized incidence rates (ASIRs), which do not account for shifts in age demographics. This limitation potentially underestimates the true increase in younger age groups.

Age–period–cohort (A-P-C) analysis offers a unique advantage by disentangling the often-confounding effects of age, period (time-related factors), and cohort (birth-year-related factors) on melanoma incidence and mortality [6,18,19,20,21]. 

This study aims to elucidate the underlying factors driving melanoma incidence trends in Spain using the GBD-2019 dataset (1990–2019). We used A-P-C analysis to investigate the independent and combined effects of age, birth cohort, and time period on melanoma incidence rates in the Spanish population. This comprehensive analysis provides valuable information on the true national burden of melanoma in Spain and informs the development of effective strategies for prevention, resource allocation, and improved patient care.

## 2. Material and Methods

This study used an ecological design to analyze trends in melanoma incidence in Spain from 1990 to 2019.

### 2.1. Data Sources

To ensure transparency and facilitate replication, annual melanoma case data for Spain (1990–2019) were obtained from the Global Health Data Exchange (GHDx) of the GBD Study 2019 (http://ghdx.healthdata.org/gbd-results-tool (accessed on 21 February 2024)). The GBD is a comprehensive initiative that gathers and synthesizes data from various sources, including national cancer registries such as Cancer Incidence in Five Continents (CI5); Surveillance, Epidemiology, and End Results (SEER); and NORDCAN. Melanoma cases were identified using International Classification of Diseases (ICD) codes: ICD-10 (C43-C43.9, Z85.82-Z85.828) and ICD-9 (172–172.9). All data were disaggregated by age and sex to enable robust analyses.

Age- and sex-specific population estimates for Spain from 1990 to 2019 were sourced from the Spanish National Statistics Institute (https://www.ine.es/ (accessed on 21 February 2024)). These estimates provide a snapshot of the resident population on 1 July each year, serving as the denominator for calculating incidence rates.

### 2.2. Statistical Analyses

To account for the nonuniform age structure of the population, age-standardized incidence rates (ASIRs) were calculated for two sexes using the direct method and the European standard population as the reference [22]. This standardization ensures that observed differences in melanoma incidence rates are not solely due to variations in population age distribution.

Joinpoint regression software version 4.9.1.0, available at https://surveillance.cancer.gov/joinpoint/ (accessed on 21 February 2024), was employed to analyze incidence trends and identify inflection points. Default settings were used to estimate the annual percentage change (APC) for each identified trend segment. Additionally, the average annual percentage change (AAPC) for the entire study period (1990–2019) was calculated using a geometrically weighted average of individual APCs, weighted by the period length. The software’s pairwise comparison function was utilized to assess similarities in trend patterns between sexes. Rates were standardized per 100,000 individuals, and male-to-female ratios were calculated.

To implement the A-P-C model, we divided the dataset into six 5-year periods (1990–1994 to 2015–2019) and 14 5-year age groups (15–19 to 80–84 years). This classification resulted in 19 birth cohorts, labeled with their median year of birth from 1910 to 2000. For each age group and period, we calculated age-specific death rates, which are crucial for the analysis of the A-P-C model.

The National Cancer Institute’s A-P-C tools (https://analysistools.nci.nih.gov/apc/ (accessed on 21 February 2024)) were used to examine A-P-C effects. The analysis included the evaluation of longitudinal age-specific rates, period/cohort rate ratios, and local/net drifts. Longitudinal age curves depicted adjusted rates within reference cohorts, while period/cohort RR’s reflected adjusted risks associated with specific periods and cohorts compared to the reference. Net drift captured the overall trend across time, while local drifts revealed trends within specific age groups. The central age, period, and cohort group served as the reference point. Wald chi-square tests were employed to assess the statistical significance of estimated model parameters. All *p*-values were two-sided, with statistical significance determined at the α = 0.05 level.

### 2.3. Ethical Considerations

Informed patient consent was not required as the data were derived from publicly available sources. This study adhered to the Guidelines for Accurate and Transparent Health Estimation Reporting for Population Health Research (GATHER), ensuring credibility and ethical integrity.

## 3. Results

An estimated 147,823 cases of melanoma were diagnosed in Spain between 1990 and 2019. The average annual growth rate was 4.6%, with a slightly higher increase in men (4.8% 95% CI, 3.7 to 5.8) compared to women (4.4% 95% CI, 3.4 to 5.3) nonsignificant).

**Figure 1:**
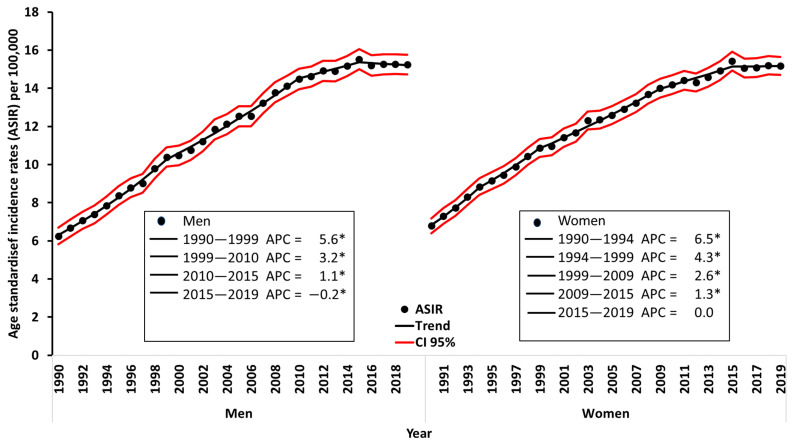
Age-standardized incidence rates (all ages) and trends estimated by joinpoint analysis for melanoma in Spain over the period 1990–2019 by sex. * indicates that the Annual Percent Change (APC) is significantly different from zero at the Alpha = 0.05 leves.

Figure 1 shows the ASIR of melanoma in Spain between 1990 and 2019, categorized by sex, together with the results of the joinpoint analysis. The graph shows a steady increase in the ASIR for both sexes, with a slightly steeper increase for men (AAPC: 2.8%, 95% CI, 2.5 to 3.2) compared to women (AAPC: 2.4%, 95% CI, 2.1 to 2.7), although this difference was not statistically significant. In 1990, the incidence rate for men was 6.2 cases per 100,000, increasing to 15.2 in 2019. For women, the rate increased from 6.8 to 15.2. Notably, the ratio between male and female incidence remained close to one throughout the period, suggesting a similar overall burden of melanoma between the two sexes. This ratio ranged from 0.9 in 1990 to 1 in 2019.

Joinpoint analysis shows similar patterns in melanoma ASIR between men and women, although the trends are not parallel. For men, the analysis shows four distinct periods: a notable increase from 1990 to 1999, with an APC of 5.6%, 95% CI, 5.1 to 6.0 (*p* < 0.05); a slowdown in the upward trend from 1999 to 2010, with an APC of 3.2% 95% CI, 3.0 to 3.4 (*p* < 0.05); a further slowdown from 2010 to 2015 (APC: 1.1%, 95% CI, 0.3 to 1.9 *p* < 0.05); and a subsequent stabilization from 2015 to 2019, with an APC of −0.2%, 95% CI, −1.0 to 0.5 (not significant). The trend of incidence for women shows four periods of decreasing growth rates (1990–1994, 1994–1999, 1999–2009, 2009–2015; APC: 6.5% (95% CI, 5.2 to 7.9), 4.3% (95% CI, 3.2 to 5.4), 2.6% (95% CI, 2.3 to 2.8), and 1.3% (95% CI, 0.8 to 1.9), respectively (*p* < 0.05), before stabilizing in a fifth period (2015–2019, APC: 0% (95% CI, −0.7 to 0.8, not significant).

**Figure 2:**
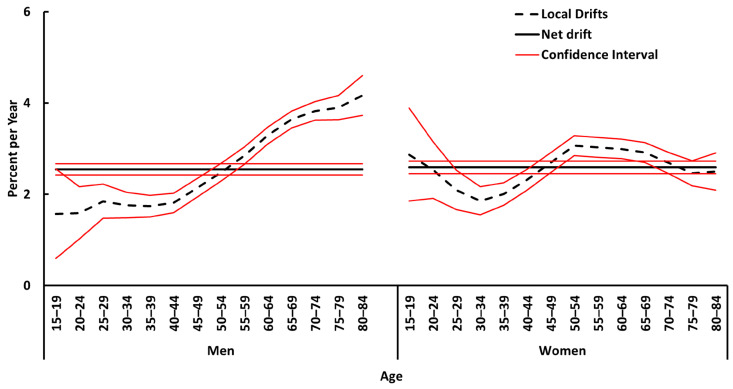
Local and net drift of melanoma incidence in Spain from 1990 to 2019, for men and women.

Figure 2 shows the net drift (annual percentage change in overall expected age-adjusted rates) and local drift (age-specific rates over time) of melanoma incidence in Spain from 1990 to 2019. The overall net drift per year was 2.5% (95% CI, 2.4% to 2.7%) for men and 2.6% (95% CI, 2.5% to 2.7%) for women. Of particular note is the increase in local drift in all age groups for both sexes from the age of 15 years.

In men, melanoma incidence rates show a more pronounced increase from younger to older age groups. During the study period, rates doubled in the older age groups compared with the younger ones. Specifically, there was a 1.6% increase in the 15–24 age group and a 4% increase in the 75–84 age group.

However, for women, the increase in rates is similar across all age groups, ranging from 1.9% in the 30–34 age group to 3.1% in the 50–54 age group.

**Figure 3:**
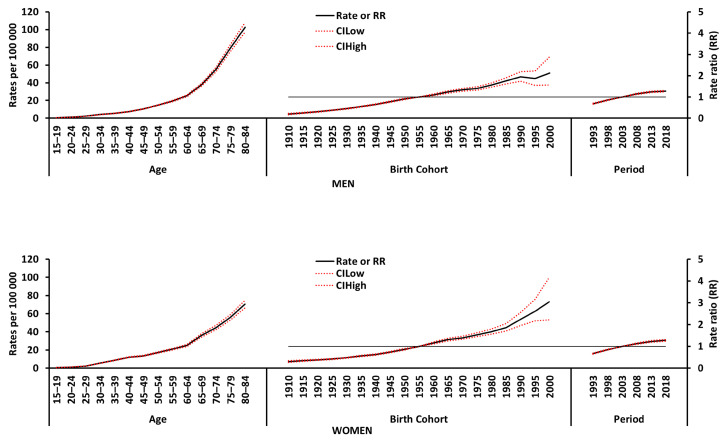
Age–period–cohort (APC) modeling results for melanoma incidence in Spain from 1990 to 2019, for men and women.

Figure 3 shows the longitudinal age curves, estimated cohort relative risk (RR) trends and estimated period RR trends for melanoma incidence in Spain by sex. Longitudinal trends (age effect) were generally consistent for both sexes, with comparable incidence risks observed in men and women. However, in younger age groups, women tended to have slightly higher rates than men, while the opposite was true in older age groups. The risk of melanoma incidence increased steadily with age in both sexes, with the highest incidence rates observed in the 80–84 age group.

The RR of melanoma incidence increased steadily in both men and women throughout the 30-year study period. The cohort RRs for melanoma incidence displayed a significant upward trend across birth cohorts for both sexes. 

Wald tests showed that all net drifts, local drifts, cohort effects, and period effects were statistically significant for both sexes (Table 1). These results indicate that the incidence of melanoma showed a statistically significant difference in local drifts and net drifts, as well as age, period, and cohort deviations.

## 4. Discussion

This study investigated the alarming increase in melanoma in Spain, employing A-P-C analysis to examine the complex influence of age, period, and birth cohort on this trend. Our findings reveal an alarming increase in melanoma rates in two sexes, and all ages (including younger populations) and generations in Spain. This trend shows no sign of slowing down. Globally, rising melanoma rates, especially in fair-skinned individuals, are largely attributed to increased sun exposure (from activities such as sunbathing and outdoor work) and improved early detection [16,18,23,24,25,26].

This trend mirrors the global patterns observed in Caucasian populations [16,18,23,24,25] and reinforces concerns highlighted by regional Spanish studies [10,27,28,29]. Interestingly, our findings contrast recent trends in northern Europe, where melanoma incidence is declining among younger populations (<70 years) [30]. This difference, along with continued increases in eastern and southern Europe (including Spain), suggests a potential shift in melanoma patterns across the continent. Although earlier detection and increased public awareness about the risks of sun exposure likely explain the deceleration observed in northern Europe [30], the factors driving the persistent rise in Mediterranean populations remain poorly understood. 

Spain’s aging population and past sun exposure practices could further exacerbate the issue [3,28]. Urgent emphasis on sun protection and screening is crucial, especially for those 50 and older, who likely experienced significant childhood sun exposure. While large-scale sun safety campaigns will benefit future generations, immediate interventions are needed [31,32,33,34]. 

Our study identifies a concerning trend: a similar rise in melanoma risk over time for both men and women, suggesting common risk factors (Figure 2). This is consistent with previous research, despite known variations across sexes and ages [10,35,36]. 

However, a key finding is the disproportionate impact on older age groups (35–64 and 65+) and the greater increase observed in men compared to women (nearly 1.5 times higher in older men) (Figure 2). Notably, unlike other countries reporting declines in younger populations [25,30,37,38], Spain continues to see rising rates among younger individuals. This potentially reflects a gap in public health efforts related to skin cancer awareness, sun protection behavior, and adapting to changing UV exposure patterns among younger generations in Spain.

Recent research sheds light on the complex factors contributing to the sex disparity in melanoma incidence: varying sun exposure patterns, knowledge and adherence to sun protection measures, and potential biological differences in skin damage and repair mechanisms between sexes [39].

To fully understand the rising trend, we used models accounting for age, period, and birth cohort effects (Figure 3).

### 4.1. Age Effect

This effect, observed in many diseases, signifies an increased risk of developing melanoma with advancing age. Our A-P-C analysis confirms this, revealing age as a strong, independent predictor of melanoma risk (Figure 3). This finding aligns with existing knowledge about the age-related decline in immune function, which can limit the body’s ability to fight malignant tumors [40]. 

### 4.2. Period Effect

Period effects, representing factors influencing disease rates across all age groups at a specific time, likely play a significant role in Spain’s rising melanoma incidence. These effects can arise from changes in various aspects, such as sun exposure habits, screening practices, or other factors affecting the entire population concurrently.

While both early-life and cumulative sun exposure contribute to melanoma risk, ultraviolet radiation remains the primary environmental culprit [41,42]. Our findings suggest a potential period effect linked to the rise in sunbathing and tanning popularity since the late 1950s and early 1960s, coinciding with Spain’s rapid economic growth.

Despite its sunny climate, Spain faces a challenge with alarmingly high sunbed use, with estimates reaching 20%—ten times higher than that of neighboring Portugal [43]. This alarming trend is further amplified by the elevated intensity of sunbed use and its prevalence among young adults and women, suggesting the need for targeted interventions for these high-risk groups. Although stricter regulations are leading to a global decline in sunbed use, individuals with a family history of melanoma demonstrate worryingly increased sunbed use and no decrease in overall sun exposure [44]. This highlights the need for more effective strategies beyond broad sun-safety campaigns to address this specific risk factor.

However, encouraging trends are emerging in sun protection behaviors, with increased sunscreen use and reduced sunburns among younger generations. While these positive developments are crucial, continued efforts are required to address the heightened risks associated with family history [44].

Compared to other cancers, melanoma’s location on the skin allows for early detection through noninvasive methods. While this can lead to an increased diagnosis rate, the early lesions are usually thin and have a better prognosis. Data from the Spanish Melanoma Registry (1997–2011) reveal encouraging trends despite the overall rise in diagnoses. The number of melanomas in situ increased significantly during these years. Excluding these melanomas, the Breslow index did not show statistically significant differences (with an increase of 0.019 mm for each current year, *p* = 0.70), while the proportion of thicker dangerous tumors remains stable [45]. This is likely due to the significant rise in detecting thinner and often more treatable tumors, including melanoma in situ. Additionally, advanced diagnostic techniques like dermatoscopy play a crucial role in identifying subtle tumors [46].

Although low-risk melanoma incidence continues to rise, improved early detection and treatment strategies are likely contributing to a decline in melanoma mortality rates in Spain [4,5,6,26]. 

Our findings highlight the crucial need for sustained and targeted educational programs that address the specific risk factors and behaviors observed in Spain. These programs should emphasize sun protection practices, encourage responsible sunbed use, and promote regular self-examinations for early detection of suspicious lesions. 

### 4.3. Cohort Effect

Our study confirms the presence of a cohort effect in melanoma, where different generations have varying risks. Although recent generations in some countries show stabilizing or even declining rates, our findings reveal they still experience higher rates compared to earlier generations. This continued rise, particularly among older adults, suggests a persistent effect from past sun exposure and limited awareness in their younger years.

While separate estimation of period and cohort effects (using period RR and cohort RR) is technically possible with specific restrictions, interpreting them independently can be challenging in real-world settings [7,21]. This difficulty arises because a single period effect, when applied to all age groups simultaneously, can manifest differently across these groups, ultimately influencing the observed cohort effect

Although the documented ASIR of melanoma in Spain has increased, it remains lower than in other European Mediterranean nations like France, Italy, Greece, and Malta [47]. This discrepancy might partially stem from the lack of a comprehensive national melanoma registry, potentially leading to an underestimation of the disease’s true burden within Spain. While regional cancer registries exist, their data indicate a 5–10% increase in invasive melanoma cases between 1990 and 2012 [48]. This regional trend aligns with observations in other European Mediterranean countries, further suggesting potential underestimation within Spain’s national data [10,27,28,29]. Patient-specific factors like skin type, red hair, and genetic variations (e.g., MC1R polymorphisms) [49] could partially explain observed differences in melanoma incidence. Environmental factors, including sunlight exposure patterns [50] and the potentially protective Mediterranean diet [51], may also play a role.

Another noteworthy consideration is the impact of Spain’s large foreign population, which mainly originates from countries with pigmented skin populations and/or lower melanoma incidence rates. In 2019, foreign-born residents in Spain constituted 9.8% of the total population, for a total number of 4,773,899 individuals (https://www.ine.es/ (accessed on 21 February 2024)). This foreign population has experienced a continuous increase in recent decades, from 1.6% (637,085 people) in 1998. This demographic aspect may contribute dynamically to the overall lower melanoma incidence observed in Spain than in its European Mediterranean counterparts.

### 4.4. Strengths and Limitations

This study has notable strengths. The robust A-P-C methodology meticulously dissects the complex interplay between age, period, and cohort effects on melanoma incidence. The inclusion of extended follow-up for older birth cohorts enhances both internal (reliability) and external (generalizability) validity, making the findings applicable to similar populations.

Our study has limitations. The Spanish melanoma data used are national estimates, susceptible to the ecological fallacy inherent in analyzed population data not always being applicable to individuals. The lack of individual-level data prevented us from analyzing how risk factors and screening practices might have changed over time. Therefore, the findings require confirmation through future individual-based studies. Additionally, while global studies like the GBD offer valuable insights, their reliance solely on modeled data can introduce potential biases and hinder a nuanced understanding of melanoma at the national level. Furthermore, our study is limited by the absence of data on melanoma subtypes, location, thickness, and stage. This information is crucial for distinguishing between a true rise in melanoma incidence and the potential increase due to diagnosing thinner, potentially less aggressive lesions, as observed in other Spanish studies [10,28]. 

The ongoing development of the GBD platform promises continuously improved data sources and modeling techniques. Therefore, this study, using the latest GBD data, provides a valuable and timely overview of melanoma incidence trends in Spain over the last three decades. Future research, incorporating data from centralized cancer registries (where established) and collaborating with dermatologists and public health officials, can address the limitations identified here. This comprehensive approach will provide deeper insights and inform the development of more targeted and effective prevention and intervention strategies to combat melanoma in Spain and worldwide.

## 5. Conclusions

By carefully examining the complex interplay of age, period, and cohort effects, this study provides valuable insights into the worrying increase in melanoma incidence in Spain. Addressing the limitations identified in this study by including individual-level data and collecting detailed information on melanoma types and risk factors will further enhance future research efforts and pave the way for the development of more targeted prevention and intervention strategies.

## Figures and Tables

**Table 1:** Wald chi-square test for estimable parameters in the age–period–cohort model.

Null Hypothesis		Men		Women	
	df	Chi-Square	*p*-Value	Chi-Square	*p*-Value
Net Drift = 0	1	1596.3	<0.001	1381.1	<0.001
All Age Deviations = 0	12	517.5	<0.001	1267.0	<0.001
All Period Deviations = 0	4	170.3	<0.001	128.2	<0.001
All Cohort Deviations = 0	17	272.4	<0.001	60.9	<0.001
All Period RR = 1	5	1611.3	<0.001	1383.4	<0.001
All Cohort RR = 1	18	3006.4	<0.001	1866.6	<0.001
All Local Drifts = Net Drift	14	269.7	<0.001	60.3	<0.001

## Data Availability

The data that support the findings of this study are openly available at: https://vizhub.healthdata.org/gbd-results/ (accessed on 21 February 2024).
